# What Elements of the Inflammatory System Are Necessary for Epileptogenesis *In Vitro*?^1^,^2^

**DOI:** 10.1523/ENEURO.0027-14.2015

**Published:** 2015-03-25

**Authors:** Kyung-Il Park, Volodymyr Dzhala, Yero Saponjian, Kevin J. Staley

**Affiliations:** 1 Department of Neurology, Massachusetts General Hospital, Boston, Massachusetts 02129; 2 Harvard Medical School, Boston, Massachusetts 02129; 3 Seoul Paik Hospital, Inje University, Seoul 100-032, South Korea

**Keywords:** epileptogenesis, immune system, inflammatory, microglia

## Abstract

Epileptogenesis *in vivo* can be altered by manipulation of molecules such as cytokines and complement that subserve intercellular signaling in both the inflammatory and central nervous systems. Because of the dual roles of these signaling molecules, it has been difficult to precisely define the role of systemic inflammation in epileptogenesis. Organotypic hippocampal brain slices can be maintained in culture independently of the systemic inflammatory system, and the rapid course of epileptogenesis in these cultures supports the idea that inflammation is not necessary for epilepsy. However, this preparation still retains key cellular inflammatory mediators. Here, we found that rodent hippocampal organotypic slice cultures depleted of T lymphocytes and microglia developed epileptic activity at essentially the same rate and to similar degrees of severity as matched control slice cultures. These data support the idea that although the inflammatory system, neurons, and glia share key intercellular signaling molecules, neither systemic nor CNS-specific cellular elements of the immune and inflammatory systems are necessary components of epileptogenesis.

## Abstract (precis)

The inflammatory and central nervous systems share many signaling molecules, compromising the utility of traditional pharmacological and knockout approaches in defining the role of inflammation in CNS disorders such as epilepsy. In an *in vitro* model of post-traumatic epileptogenesis, the development of epilepsy proceeded in the absence of the systemic inflammatory system, and was unaffected by removal of cellular mediators of inflammation, including macrophages and T-lymphocytes.

## Significance Statement

The inflammatory and central nervous systems share many signaling molecules, compromising the utility of traditional pharmacological and knockout approaches in defining the role of inflammation in CNS disorders such as epilepsy. In an *in vitro* model of post-traumatic epileptogenesis, the development of epilepsy proceeded in the absence of the systemic inflammatory system, and was unaffected by removal of cellular mediators of inflammation, including macrophages and T-lymphocytes. These results are not meant to disprove the idea that “inflammation causes epilepsy”, but rather circumscribe the overlap between the inflammatory system versus the CNS mechanisms that are operative during post-traumatic epileptogenesis.

## Introduction

Local and systemic inflammation may play a role in epileptogenesis (Vezzani et al., 2012). The brain is largely shielded from the systemic immune system by the blood−brain barrier (Lampron et al., 2013), but an active innate immune system converges to activate phagocytic final effectors, the microglia. These cells comprise 10% of the cells in the brain (Benarroch, 2013), where lymphocytes are also present (Ravizza et al., 2008).

There is robust pathological evidence for the involvement of cellular elements of the immune and inflammatory systems, including T lymphocytes and microglia, in epilepsy syndromes such as Rassmussen’s encephalitis (Bien et al., 2002; Granata and Andermann, 2013). Other epilepsy syndromes are driven by humoral elements of the immune system, including antibodies to NMDA receptors and other neuronal proteins (Davis and Dalmau, 2013; Gresa-Arribas et al., 2014). Inflammatory mediators, including the cytokines interleukin (IL)-6 and IL-1β, complement cascade factor C1q, transforming growth factor (TGF)-β, and tumor necrosis factor alpha (TNFα), are upregulated in human epileptic tissue (Vezzani et al., 2012; Liimatainen et al., 2013). These inflammatory mediators are increased experimentally by prolonged seizures (Minami et al., 1991; Vezzani et al., 1999). Manipulation of immune and inflammatory mediators alter seizures. Inhibition of leukocyte infiltration of the blood−brain barrier prevented experimental epilepsy (Fabene et al., 2008), and IL-1β antagonists reduced induced seizures (Librizzi et al., 2012).

The interpretation of these intriguing findings is complicated by two issues. First, these inflammatory mediators also play important roles in physiological synaptic modifications, including those that underlie learning and memory. The cytokines implicated in epilepsy are also produced by neurons and astrocytes in the normal, uninflamed brain (Vitkovic et al., 2000; Yirmiya and Goshen, 2011; Pribiag and Stellwagen, 2014). For example, IL-1β and IL-6 are increased by synaptic stimuli that induce physiological synaptic plasticity (Schneider et al., 1998; Balschun et al., 2004). Cellular and cytokine elements of the immune and inflammatory systems, including T lymphocytes and IL-1β, play essential roles in learning and memory (Schneider et al., 1998; Yirmiya et al., 2002; Kipnis et al., 2004). Homeostatic scaling of synaptic strength entails cytokines including TNFα, and possibly IL-1β (Pribiag and Stellwagen, 2014). Microglia participate in physiological anatomical synaptic alterations (Paolicelli et al., 2011; Schafer et al., 2012). Microglial activation after epileptogenic injuries is complex and not strongly correlated with neuronal loss (Papageorgiou et al. 2014). Kindling studies have not found significant increases in microglia or cytokines (Khurgel et al., 1995; Tooyama et al., 2002; Aalbers et al., 2014), and experimental antiepileptogenic therapies are not associated with changes in microglial activation (van Vliet et al., 2012). Recent studies exploring the expression of cytokines in temporal lobe epilepsy have not found evidence that inflammation is a necessary for element of hippocampal sclerosis (Aalbers et al., 2014).

Second, inflammation and cell loss coexist (Tooyama et al., 2002). The clinical and experimental situations in which the inflammatory system has been implicated in epileptogenesis are also characterized by neuronal death and astrogliosis (Ravizza et al., 2008). Neuronal death and astrogliosis are also epileptogenic (Dudek and Staley, 2012), although it has not been demonstrated whether inflammation and cell loss act independently.

Studying the relation of inflammation and epilepsy by removal of elements of the systemic inflammatory system *in vivo* is impractical due to ongoing monocytic immigration from the bloodstream (Ransohoff and Cardona, 2010). As a proof-of-concept experiment to test the necessity of the immune and inflammatory systems in epileptogenesis, we used a reduced *in vitro* system in which the cellular effector elements of these systems can be removed and the effects on epileptogenesis observed. The hippocampal organotypic slice culture is a well-characterized *in vitro* system of epileptogenesis (McBain et al., 1989; Gutnick et al. 1989; Bausch and McNamara, 2000; Dyhrfjeld-Johnsen et al., 2010; Marchi et al., 2011; Berdichevsky et al., 2012, 2013; Ransohoff and Brown, 2012; Albus et al. 2013). Blood vessels are present but the blood−brain barrier is not operative and the systemic immune system is not present. We characterized epileptogenesis in preparations from control and T lymphocyte-deficient nude mice, with and without microglial depletion by clodronate (Kohl et al. 2003; Kumamaru et al., 2012).

## Materials and Methods

### Culture of organotypic hippocampal slices

All animal-use protocols were in accordance with the guidelines of the National Institutes of Health and our Institution for Comparative Medicine on the use of laboratory animals, and approved by the Subcommittee on Research and Animal Care. Hippocampal slices were prepared at postnatal days 6-8 from C57BL/6 mice or T lymphocyte-deficient nude mice (Foxn1nu/Foxn1nu, http://jaxmice.jax.org/strain/002019.html) or Sprague-Dawley rats of either sex. Slices (400 μm thick) from a McIlwain tissue chopper (Mickle Laboratory Engineering Co.) were mounted in clots of chicken plasma (Cocalico Biologicals) and thrombin (Sigma-Aldrich) on poly-L-lysine-coated glass coverslips (Electron Microscopy Sciences). Slices were incubated in roller tubes (Nunc) at 37 °C within 750 μl of NeurobasalA/B27 medium supplemented with 0.5 mM GlutaMAX and 30 μg/ml gentamicin (Invitrogen). This concentration of gentamycin was less than half the lowest concentration demonstrated to induce regenerative activity *in vitro* (Grøndahl and Langmoen 1993). Culture media were changed biweekly.

### Field potential recording

Extracellular field potentials were recorded in area CA1 or CA3 of hippocampal slices in a conventional submerged chamber using tungsten-coated microelectrodes (diameter, 50 μM). Oxygenated (95% O_2_ and 5% CO_2_) artificial CSF containing 126 mM NaCl, 3.5 mM KCl, 2 mM CaCl_2_, 1.3 mM MgCl_2_, 25 mM NaHCO_3_, 1.2 mM NaH_2_PO_4_, and 11 mM glucose (pH 7.4), were continuously perfused at 33 ± 0.5 °C. Flow rate was 2.5 ml/min. Before actual recording, slices were allowed to stabilize in the recording chamber for at least 1 h, and then extracellular field potentials were recorded for 1 h in each slice. pCLAMP 8.2 (Axon Instruments) was used for data acquisition. Recordings were sampled at 10 kHz and filtered from 1 Hz to 1 kHz. Seizure-like activity (SLA) was defined as high amplitude (×3 baseline), high frequency (>10 Hz) spikes followed by afterdischarges, with the duration of the spike and afterdischarge complex lasting more than 5 s. Incidence of SLA and numbers of slices and animal in the experiment are summarized in Table 1.

**Table 1.** Numbers of slices and animals used in each experiment

Recording timing	Slice numbers (per group)	Frequency of seizure (/h)		Number of animals used	Incidence of epilepsy (%)	
		Clodronate	Control		Clodronate	Control
Figure 2						
DIV12	11	1-36	0-24	3 rats	100	72.7
Figure 3						
DIV6	6	0-2	0-2	2 rats	83.3	50
DIV12	6	0-12	0-3	2 rats	83.3	66.7
DIV22	8	0-3	0-2	2 rats	37.5	25.0
Figure 4						
DIV6	4-5	9-19	1-16	2 mice	100	100
DIV12	4-6	2-26	2-21	2 mice	100	100
Figure 5						
DIV12	4-6	0-2	0-4	3 nude mice	40.0	62.5

### Chemical depletion of microglia

Clodronate is a selective macrophagic and microglial toxin without evidence of injury to other cellular components (Kohl et al. 2003; Kumamaru et al., 2012). Perivascular macrophages could be retained in slice cultures, but these cells are also depleted by clodromate at concentrations below those needed to deplete microglia (Polfliet et al., 2001). To assess whether microglia alter the course of epileptogenesis or ictogenesis, we incubated slice cultures in clodronate-encapsulated liposomes (Clodrosome, purchased at www.clodrosome.com, containing 17 mM of clodronate disodium salt, 24 mM of phosphatidylcholine, and 11 mM of cholesterol suspended in PBS) for 3-6 d, as specified in the Results (Fig. 4*A*). During these incubations, Clodrosome was replenished with each media change.

### Immunohistochemistry of organotypic hippocampal slices

Slices were fixed overnight with 4% paraformaldehyde at room temperature. They were washed with PBS several times and stored in PBS at 4 °C until use. The hippocampi were scraped out gently from the bottom of petri dishes or coverslips and then treated with 0.5% Triton X-100 overnight. Blocking was done with 20% bovine serum albumin (BSA) for 4 h at room temperature on a shaker. We incubated tissues overnight with the microglial-specific antibody anti-ionic calcium-binding adapter molecule 1 (Iba-1, 1:1000; Waco Chemicals), a protein present in both perivascular macrophages and microglia (Sasaki et al. 2001), with/without the neuron-specific antibody anti-NeuN (1:50; Millipore) as primary antibodies. Goat anti-rabbit IgG Alexa 594 (1:200; Invitrogen) with/without goat anti-mouse IgG Alexa 405 (1:200; Invitrogen) were used as secondary antibodies. All antibodies were dissolved in 5% BSA. DAPI (300 nM; Sigma-Aldrich) was added for nuclear counter-staining in some slices. Imaging was done using a two-photon microscope (Olympus Optical) with 20× water-immersion objective. For cell counts, the pyramidal layer of CA1 was centered on the *y*-axis of the microscopic field. The *x*-coordinates of the field were determined by centering an imaginary line perpendicular to the pyramidal layer that passed through the middle of the superior blade of dentate gyrus. Ten serial *xy* planes images with best intensities were acquired at 2 μm vertical (*z*) intervals in the CA1 subfield. Cell counting for NeuN(+) or Iba-1(+) was performed in Z-stack images from whole microscopic field using ImageJ software.

### Lactate and LDH assays of clodronate effects

Organotypic hippocampal slice cultures from C57BL/6 mice were cultured in poly-D-lysine-coated six-well tissue culture plates. Three slices were treated with clodronate-encapsulated liposome (final concentration: 0.2 mg/ml) and three slices were treated with an equal concentration of empty liposome from DIV0-6. All six slices were prepared from the same animal. Following a thorough wash-out on DIV6, culture medium with PBS was applied to both groups of slices from DIV6 to DIV28. Culture media were collected and changed biweekly. Images of all slices were obtained at media changes via brightfield microscopy with an Olympus CKX41 inverted microscope. Lactate was used as a biomarker of ictal activity (Berdichevsky et al. 2013) and lactate dehydrogenase (LDH) concentrations were used as an assay of cell death (Berdichevsky et al. 2012), measured in spent culture medium collected during the biweekly media changes.

### Statistical analyses

All values are expressed as mean ± SEM. Statistical significance were evaluated with Student *t* tests without or with Sidak correction for multiple comparisons where indicated, Fisher’s exact test, or one-way/two-way ANOVA as indicated. Alpha *p* value < 0.05 is considered to be significantly different.

## Results

### Clodronate depletes microglia in organotypic slices

**Figure 1.**
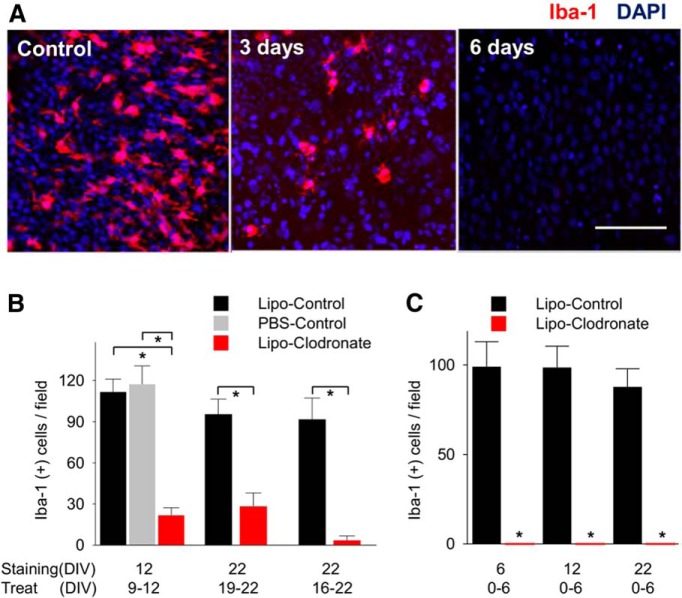
Elimination of microglia using liposomal (Lipo) clodronate from organotypic hippocampal slices of rat**. *A,*** Iba-1-positive cells (microglia and macrophages) in CA1 were depleted by using liposomal clodronate (0.2 mg/ml). ***B,*** Treatment for 3 d (from DIV9 to 12) decreased the number of microglia comparing liposome-control group and saline control group. Six day treatment from DIV16 to DIV22 eliminated more Iba-1(+) cells than 3 d treatment (96.2% vs 70.4%, *n* = 4 per group, *p* = 0.07). ***C,*** Microglial depletion persisted 16 d after washout. All values are expressed as mean ± SEM. *p < 0.05. Scale bar, 100 µm.

The concentration and duration of clodronate treatment in organotypic slices was optimized to obtain maximal microglial depletion. An equal concentration of empty liposomes was used as the control condition in all experiments utilizing clodronate. When clodronate (final concentration of 0.2 mg/ml in the culture media) was applied for 3 d to rat slices from DIV16 to DIV19, the number of microglia counted in the CA1 subfield decreased by 70.4% (95.5 ± 10.9 vs 28.25 ± 9.8 per field; *n* = 4; *p* = 0.004^a^; Table 2; Fig. 1*B*). The remaining microglia were small and rounded in shape, and had few ramified processes (Fig. 1*A*). To increase microglial depletion, the duration of treatment was extended up to 6 d (DIV16−DIV22). This reduced microglia by 96.2% (91.8 ± 15.5 vs 3.5 ± 3.2; *n* = 4; *p* = 0.001^b^; Fig. 1*B*) compared to controls. The number of Iba-1(−) cells was not different between groups (320.3 ± 8.6 per field in clodronate group vs 325.5 ± 10.3 per field in control group, *n* = 4, *p* = 0.77^c^). The number of CA1 microglia in control slices treated with empty liposomes was not different from slices treated with an equal volume of PBS. Following clodronate depletion, microglia were not regenerated even after 3 weeks in culture (Fig. 1*C*), indicating that clodronate also effectively eliminated microglial progenitor cells.

**Table 2.** Statistical table

	Data structure	Type of test	Power
a	Normally distributed	*t* test	0.004
b	Normally distributed	*t* test	0.001
c	Normally distributed	*t* test	0.77
d	Categorical	Fisher’s exact test	0.07
e	Normally distributed	One-way ANOVA	0.02
f	Normally distributed	One-way ANOVA	0.11
g	Normal distributed	One-way ANOVA	0.14
h	Categorical	Fisher’s exact test	0.24
i	Categorical	Fisher’s exact test	0.52
j	Categorical	Fisher’s exact test	0.60
k	Normally distributed	Two-way ANOVA	0.39 for frequency, 0.46 for total duration, and 0.30 for mean duration
l	Normally distributed	One-way ANOVA	0.31
m	Normally distributed	One-way ANOVA	0.36
n	Normally distributed	One-way ANOVA	0.56
o	Normally distributed	One-way ANOVA	0.99
p	Normally distributed	One-way ANOVA	0.89
q	Normally distributed	One-way ANOVA	0.62
r	Normally distributed	*t* test	0.003
s	Normally distributed	*t* test	0.01
t	Categorical	Fisher’s exact test	0.36
u	Normally distributed	One-way ANOVA	0.54
v	Normally distributed	One-way ANOVA	0.051
w*	Normally distributed	*t* test with Sidak correction	0.02, 0.42, 0.08, 0.07, 0.09, 0.08, 0.08, and 0.13 at each time point
x*	Normally distributed	*t* test with Sidak correction	0.10, 0.02, 0.001, 0.002, 0.002, 0.002, 0.007, 0.01 at each time point

*α_SID_ = 1 − (1 − α)^1/^*^m^*, α = 0.05, *m* = 1,2,3,4,5,6,7,8. α_SID(MAX)_ = 0.06

### Microglial depletion has no anticonvulsant effects in rat slices

**Figure 2.**
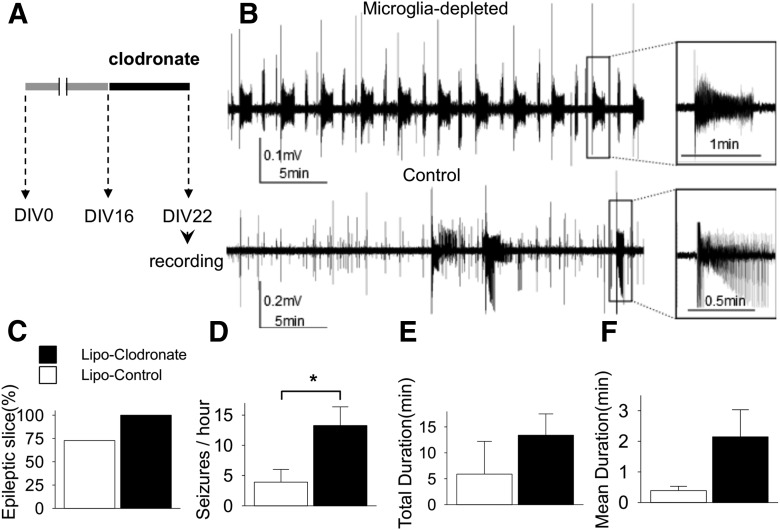
Effect of microglial depletion on ictogenesis in cultured rat slices. ***A,*** Schematic drawing of experiment protocol. ***B,*** Representative traces of field potentials recorded at DIV22 show that the microglia-depleted slice had more frequent and longer seizure-like activities compared to the control slice. ***C,*** Bar graphs indicate the percentage of slices with more than one seizure-like activity during observation period in each group. ***D−F,*** The frequency of seizure-like activities was significantly greater in the microglia-depleted group (13.3 ± 3.1 vs 3.9 ± 2.1/h, *n* = 11 per group, *p* = 0.02). All values are expressed as mean ± SEM, *p < 0.05.

We compared spontaneous SLAs at DIV22 according to the presence or absence of microglia (*n* = 11 each group). Equal numbers of slices from each animal were allocated to clodronate or control groups. Extracellular field potentials were recorded for 1 h in each slice. All slices showed at least one SLA in the microglia-depleted group and only 72.7% showed SLA in the control group (*p* = 0.07^d^; Fig. 2*C*). The frequency of SLA in microglia-depleted slices was significantly higher than control (13.3 ± 3.1 vs 3.9 ± 2.1 SLA/h; *p* = 0.02^e^; Fig. 2*D*). Total duration (13.4 ± 4.1 vs 4.3 ± 3.5 min; *p* = 0.11^f^; Fig. 2*E*) and mean duration of each SLA (2.1 ± 0.9 vs 0.5 ± 0.2 min; *p* = 0.14^g^; Fig. 2*F*) were longer in microglia-depleted slices than control, but the differences did not reach statistical significance.

### Microglial depletion has no antiepileptogenic effects in rat slices

**Figure 3.**
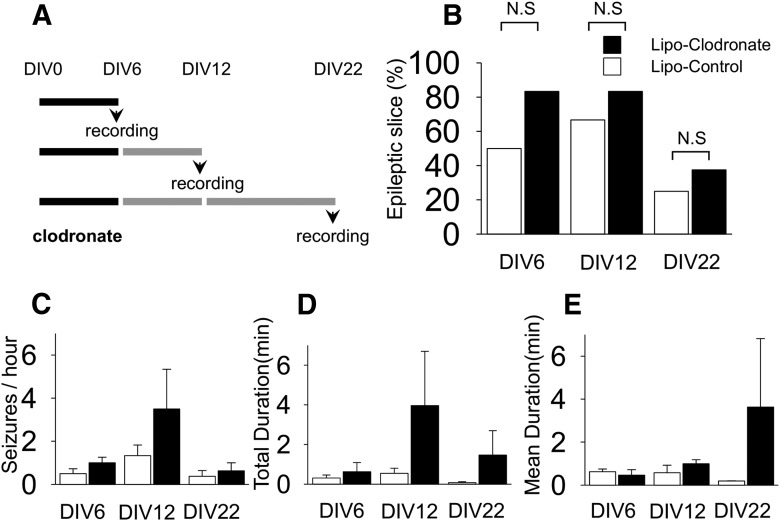
Effect of microglial depletion on epileptogenesis in cultured rat slices. ***A,*** Liposomal (Lipo) clodronate or liposome-control was exposed to slices from DIV0 to 6 and spontaneous seizure-like activities were recorded at DIV6, 12, or 22 according to the indicated protocols. ***B,*** The proportions of slices demonstrating seizure-like activity during recording were not different between microglia-negative group and control group (*n* = 6-8 per group, *p* = 0.24, 0.52, and 0.60. respectively). ***C−E,*** Seizure frequency, total recorded seizure time, and mean seizure duration tend to be higher in slices depleted of microglia, although none of these differences were statistically different (*n* = 6-8 per group, *p* = 0.24, 0.30, and 0.28, respectively). N.S, Not significant. All values are expressed as mean ± SEM.

The preceding experiments indicate that microglial depletion had no anticonvulsant effect on seizure activity in cultured slices in which epilepsy was established. To evaluate the role of microglia on epileptogenesis, we applied clodronate before the onset of seizures, that is, from the beginning of culture (DIV0) to DIV6. In the organotypic hippocampal slice model, this time period is considered as the “latent” period, i.e. the time between brain injury and the development of spontaneous seizure activity (Berdichevsky et al., 2012). Following microglial depletion, field potentials were recorded at DIV6, 12, and 22 (Fig. 3*A*). SLAs were observed in 83.3% (vs 50% in control, *n* = 6 each group, *p* = 0.24^h^), 83.3% (vs 66.7% in control, *n* = 6 each group, *p* = 0.52^i^), and 37.5% (vs 25.0% in control, *n* = 8 each group, *p* = 0.60^j^) in microglia-depleted slices on recoding at DIV6, 12, and 22, respectively. Overall, the proportions of epileptic slices, which were defined as having more than one seizure during the observation time, were not statistically different between the microglia-depleted and control groups (Fig. 3*B*). The values for SLA frequency, duty cycle, and duration are listed in Table 3. On recording at DIV6 and 22, there were no significant differences in these parameters between microglia-depleted and control slices. These three parameters tended to be increased in microglia-depleted slices at DIV12, but the differences did not reach significance between the groups (Fig. 3*C−E*). Overall, there were no statistical differences between groups or ages^k^.

**Table 3.** Comparison of seizure-like activities between microglia-depleted and control slices

	Microglia-depleted	Control	*p* value
Recording at DIV6			
Frequency (SLA/h)	1.0 ± 0.3	0.5 ± 0.2	0.17
Total duration (s/h)	37.7 ± 27.7	18.7 ± 9.0	0.53
Mean duration (s)	27.7 ± 15.3	37.3 ± 7.7	0.67
Recording at DIV12			
Frequency (SLA/h)	3.5 ± 1.8	1.3 ± 0.5	0.24
Total duration (s/h)	237.7 ± 164.2	32.8 ± 15.3	0.30
Mean duration (s)	59.6 ± 11.1	34.5 ± 20.9	0.28
Recording at DIV22			
Frequency (SLA/h)	0.6 ± 0.4	0.4 ± 0.3	0.59
Total duration (s/h)	88.0 ± 73.8	4.4 ± 3.1	0.28
Mean duration (s)	217.8 ± 191.1	11.5 ± 0.5	0.46

### Microglial depletion has no anticonvulsant or antiepileptogenic effects in mice

**Figure 4.**
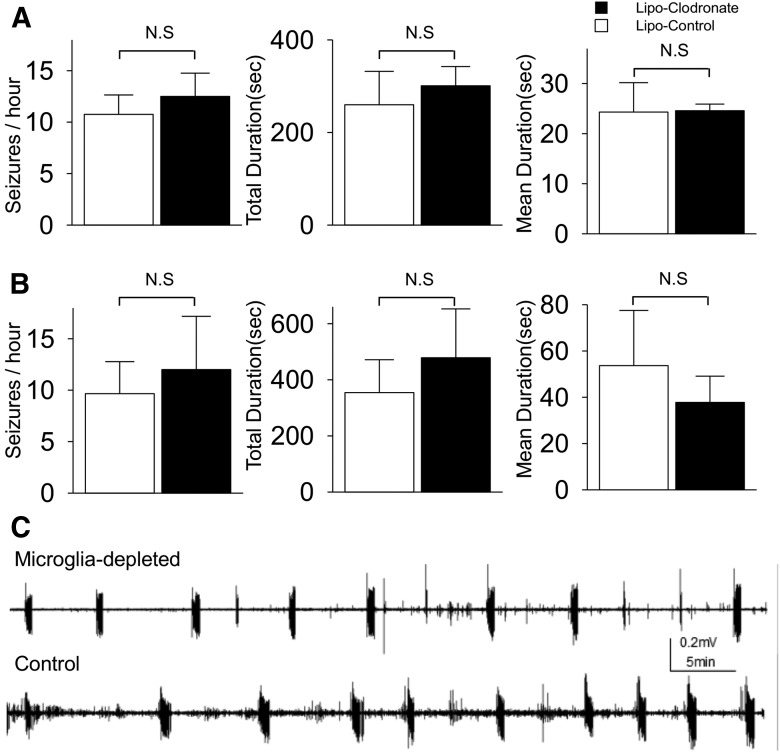
Effect of microglial depletion on epileptogenesis and ictogenesis in wild-type mice slices. ***A,*** Data was recorded at DIV6 after exposure of liposome clodronate from DIV0 to 6. ***B,*** Data was recorded at DIV12 after exposure of liposomal clodronate from DIV6 to 12. Microglial depletion did not alter the frequency, total duration, or mean duration of seizure-like activities (*n* = 4-5 per group). ***C,*** Representative traces recorded at DIV6 from microglia-depleted and control group, shows similar patterns of spontaneous seizure-like activities. All values are expressed as mean ± SEM. N.S, Not significant.

Organotypic slices from wild-type C57BL/6 mice usually demonstrated more frequent SLA compared with age-matched rat slices. To test the generalizability of the results from rat slices, we repeated the experiments described above in murine hippocampal slice cultures. Depletion of microglia by clodronate exposure from DIV0 and 6 did not alter spontaneous SLAs recorded at DIV6 (Fig. 4*A*). SLA frequency was 12.5 ± 2.3 per hour in microglia-depleted slices (*n* = 4) and 10.8 ± 1.9 per hour in control slices (*n* = 5) (*p* = 0.31^l^). Other parameters—total duration (5.0 ± 0.7 min vs 4.3 ± 1.2 min; *p* = 0.36^m^) and mean duration (24.6 ± 1.3 s vs 24.3 ± 5.9 s; *p* = 0.56^n^)—in microglia-depleted slices were also similar to control. Next, we applied clodronate from DIV6 to DIV12 and recorded at DIV12 to assess the effect of microglial depletion on ictogenesis. The frequency was 12.0 ± 5.2 per hour for microglia-depleted (*n* = 4) and 9.7 ± 3.1 per hour for control (*n* = 6) (*p* = 0.99^°^). Total SLA duration and mean SLA duration were 8.0 ± 2.9 min, 37.8 ± 11.3 s for microglia-depleted and 5.9 ± 1.9 min, 53.7 ± 23.8 s for control, respectively, and these differences were not statistically significant (*p* = 0.89^p^ for total duration, *p* = 0.62^q^ for mean duration). We conclude that microglial depletion at these time periods in murine organotypic hippocampal slice cultures do not alter epileptogenesis or ictogenesis.

### Epileptogenesis is not dependent on T lymphocytes and/or microglia

**Figure 5.**
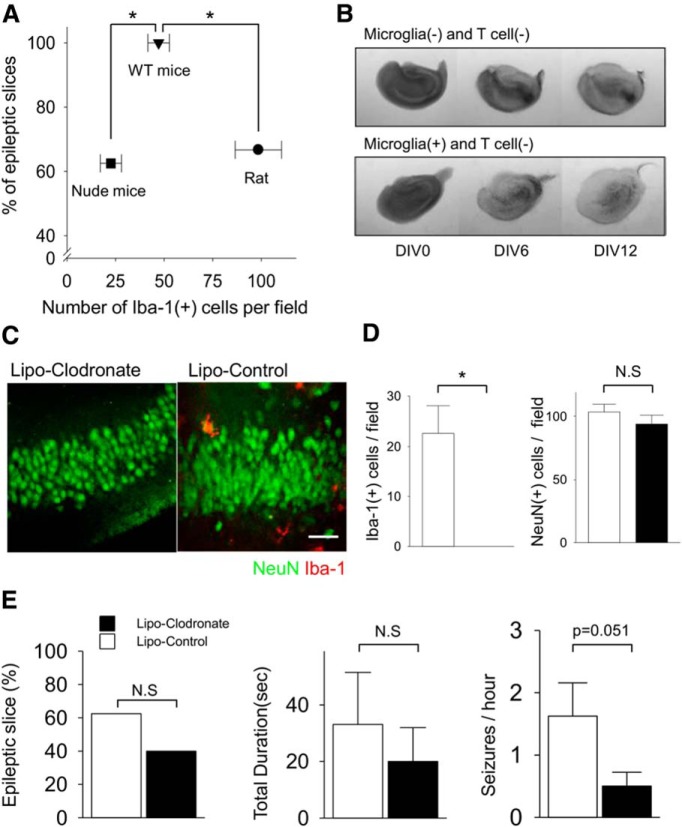
Microglial depletion from slices of nude mouse. ***A,*** Comparison of the density of Iba1-positive cells in area CA1 in control slices from different species (*n* = 5-6 per group) versus the percentage of slices that displayed seizure activity (*n* = 5-8 per group; different slices used for Iba-1 staining and recording). Across species, there was no significant correlation between microglial density and fraction of epileptic slices (*R* = −0.10, *p* = 0.94). ***B*, **Examples of hippocampal cultures from nude mice at each time point. Liposomal (Lipo) clodronate (top, 0.02 mg/ml) or liposome-control (bottom) was applied from DIV0 to 6. ***C, D,*** Double immunostaining was performed with NeuN and Iba-1 antibodies. Quantification at CA1 reveals that liposomal clodronate did not affect the neuronal populations of nude mouse (*p* = 0.33), whereas it depleted all microglia (*n* = 5 per group). ***E,*** The proportion of epileptic slices and total duration of seizure-like activities in microglia-depleted slices did not differ significantly from control slices, whereas the frequency of seizure-like activity was somewhat lower in microglia-depleted slices (*n* = 8-10 per group). All values are expressed as mean ± SEM. *p < 0.05; N.S, not significant. Scale bar, 100 μm.

T lymphocytes are a part of the adaptive immune system that play an important role in activating the innate immune, i.e. inflammatory system, of the brain (Ransohoff and Brown, 2012) and have been hypothesized to contribute to epileptogenesis (Fabene et al. 2008; Marchi et al., 2011). To investigate the role of this cellular component of the immune system on epilepsy more precisely, we compared wild-type organotypic slices to organotypic slices depleted of either T lymphocytes or both microglia and T lymphocytes by treating slices prepared from nude mice with clodronate (0.02 mg/ml). The numbers of microglia were counted in area CA1 in the three different preparations (slice cultures from wild-type (WT), nude (T lymphocyte-deficient) mice, and slices from nude mice treated with clodronate, *n* = 5-6 each group). Nude mice showed fewer microglia per field (*p* = 0.003^r^ vs rat, *p* = 0.01^s^ vs WT mouse; Fig. 5*A*). However, there was no correlation between the density of microglia and the incidence of epileptic slices across species (Fig. 5*A*). In mice, a lower concentration (0.02 mg/ml) of clodronate eliminated microglia as effectively as the higher concentration used in rat slices (0.2 mg/ml), so we used the lower concentration in the following experiments. Clodronate at 0.02 mg/ml did not alter neuronal populations (Fig. 5*C*,*D*). Epileptogenesis proceeded at a similar rate in clodronate-treated nude mouse slices as in control slices. Recordings at DIV12 demonstrated SLAs in 40.0% of double-deficient slices (*n* = 10) versus 62.5% in control slices prepared from nude mice (*n* = 8) (*p* = 0.36^t^). Analysis of seizure parameters (Fig. 5*E*) revealed that the total duration of SLAs in clodronate-treated nude mouse slices was not different from that of control slices (20.0 ± 12.0 s vs 33.1 ± 18.4 s; *p* = 0.54^u^). SLAs were rarely observed in both groups. However, the frequency of SLAs in the double-deficient preparations was decreased compared to controls (0.5 ± 0.2 SLAs/h vs 1.6 ± 0.5 SLAs/h; *p* = 0.051^v^). Thus, we conclude that epileptogenesis is not dependent on T lymphocytes and/or microglia in this model.

### The time course of epileptogenesis and ictal cell death are not altered by the absence of microglia

**Figure 6.**
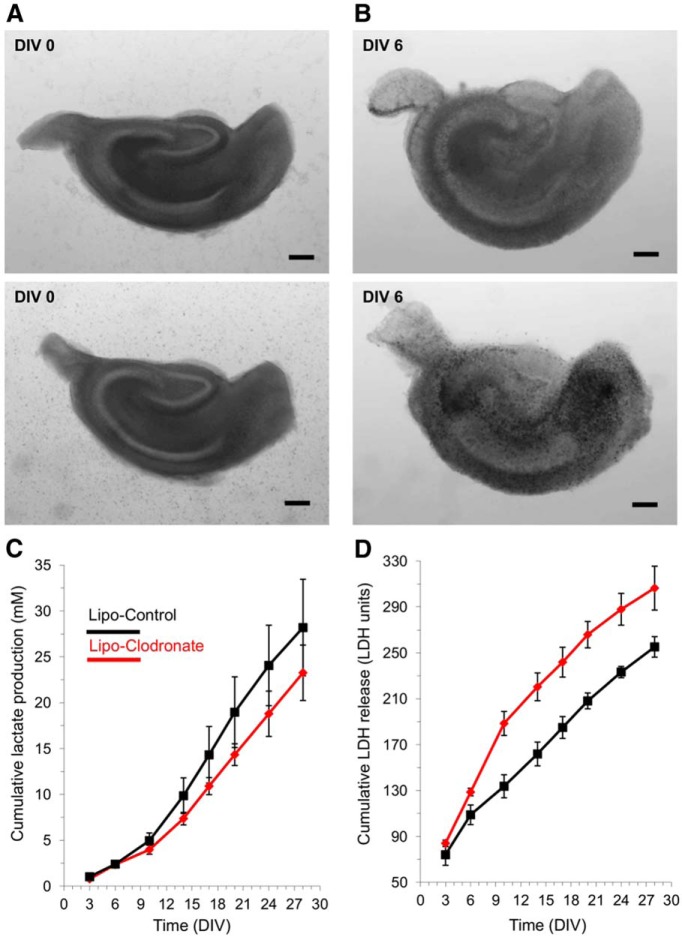
Long-term assays of epileptogenesis in microglia-depleted versus control hippocampal slice cultures. ***A,*** Examples of slice culture brightfield micrographs at DIV0 prior to clodronate treatment of upper slice. ***B,*** Brightfield micrographs of the same slice cultures on DIV6 at the conclusion of clodronate treatment to the upper slice and empty liposome treatment of the lower slice. No deleterious effects of clodronate are evident at this magnification. ***C,*** Cumulative group mean lactate production, assayed in the spent culture media at 3-4 daintervals during twice weekly media changes. *N* = 3 slices each group; all slices from the same animal. ***D,*** Cumulative group mean LDH release, assayed in the spent culture media. Same groups slices and media as for lactate assays in panel C. All values are expressed as mean ± SEM. Scale bar, 250 μm.

As epileptogenesis progresses in organotypic slices, more SLAs are generated (Dyhrfjeld-Johnsen et al., 2010), and just as in human epilepsy (Lazeyras et al., 2000; Canas et al., 2010), the increased epileptiform activity is associated with increases in local lactate (Berdichevsky et al., 2013). Ictal excitotoxic neuronal injury releases the cytoplasmic LDH to the culture media (Berdichevsky et al., 2012, 2013). To be certain that we had not missed a critical compensation that normalized epileptogenesis in the slices depleted of microglia, we used the lactate and LDH assays to follow the detailed time course of epileptogenesis *in vitro*. Mouse hippocampal slices were exposed to clodronate (0.2 mg/ml) at DIV0-6 (Fig. 6*A*,*B*), and lactate and LDH levels were assayed at each subsequent media change in three depleted versus three control slices from the same animal. Cumulative lactate production was slightly reduced in microglial-deficient slices (Fig. 6*C*), but this reduction was in line with the 10% reduction in total cell number due to microglial depletion (Benarroch, 2013), did not reach statistical significance^w^ at any time point, and was much less than the lactate reduction during anticonvulsant treatment (Berdichevsky et al., 2012). Cumulative LDH release was increased early in the clodronate-treated slices (Fig. 6*D*), consistent with microglial cell death, but this difference was not sustained statistically^x^.

## Discussion

In this *in vitro* study of post-traumatic epileptogenesis, electrophysiological recordings as well as lactate and LDH assays were not significantly different in slices depleted of either microglia or both T lymphocytes and microglia. We conclude that the systemic immune system, a compromised blood−brain barrier, and key cellular elements of the immune and inflammatory systems, including T lymphocytes and microglia, are not necessary for epileptogenesis, at least in this *in vitro* model.

### Limitations

This study utilized a well-characterized *in vitro* model of epileptogenesis, the rodent hippocampal organotypic slice preparation. Our goal was to test whether cellular effectors of inflammation were necessary elements of epileptogenesis, so we did not attempt to quantify the number of T lymphoctyes present in the slices from wild-type versus nude mice, nor did we attempt to quantify the relative effects of T lymphocyte depletion on the rate of epileptogenesis. In this *in vitro* preparation, the control slices are also separated from the systemic immune system. Thus, we are not able to resolve whether epileptogenesis might proceed at a more rapid rate if systemic elements of the immune system were interacting across the blood−brain barrier (Fabene et al., 2008). However, the cell loss (Berdichevsky et al., 2012), inflammatory reaction (degree of microglial activation in Fig. 1*A*), and rate of epileptogenesis (Dhyrfjeld-Johnesen et al., 2010; Berdichevsky et al., 2012) in the organotypic slice culture matches or exceeds what is observed *in vivo* (Tooyama et al., 2002; Ravizza et al., 2008; Williams et al., 2009; van Vliet et al., 2012), so there is no reason to expect that inflammation, epileptogenesis, or ictogenesis has been compromised in the control slices.

The epileptic activity that develops in organotypic slices is severe: although SLA can be temporarily suppressed with phenytoin, SLA recrudesces after 3 weeks in culture (Berdichevsky et al., 2012). Although this recrudescence parallels human post-traumatic epilepsy (Temkin et al., 1990; Temkin, 2009), it is possible that a less severe epilepsy model would reveal modulatory effects of immunity or inflammation more readily than the organotypic slice culture model. Thus, we cannot conclude from these experiments that the immune and inflammatory systems are not involved in all epileptogeneses, but only that key cellular elements of these systems are not necessary for post-traumatic epileptogenesis, and that so far, this has only been demonstrated *in vitro*.

We did observe differences in epileptogenesis between murine slices from C57BL/6 mice (Fig. 4) versus slice cultures from nude mice (Fig. 5). However, inbred mouse strains have variable rates of epileptogenesis (Papandrea et al., 2009), wild-type controls are not available for nude mice, and epileptogenesis in slices from nude mice were comparable to slices from rats (Fig. 5A). Thus, we can deduce that epileptogenesis proceeds in the absence of T lymphocytes, but we cannot make conclusions regarding potential influences of T lymphocytes on the rate of epileptogenesis. Overall, there was no correlation between microglial counts and the fraction of epileptic slices across species (Fig. 5*A*). In slices from animals other than nude mice, the increased seizure frequency after microglia depletion may reflect the neuroprotective actions of microglia (Mosser and Edwards, 2008; Smith et al., 2012; Benarroch, 2013), although the magnitude of effects were not consistent between strains and species. These minor differences in epileptogenesis do not diminish the importance of the central finding that slices without cellular mediators of inflammation exhibit robust epileptogenesis. This implies that the full inflammatory cascade cannot be a necessary component of all forms of epileptogenesis.

Because microglia were progressively eliminated from 0 to 6 d after trauma, our experiments do not preclude a necessary but very early role for microglia in epileptogenesis. However, manipulation of inflammatory mediators during this interval are ineffective *in vivo* (Noe et al., 2013), indicating that if inflammation had a necessary role in epileptogenesis, it would extend beyond the phase during which the slice cultures still contained microglia.

Although *in vitro* experiments must be extrapolated with caution, it should also be born in mind that there is no feasible way to remove the blood−brain barrier, systemic immune system, and local cellular elements of the immune and inflammatory systems from current *in vivo* models.

### Implications

Determining whether a particular system is involved in a mechanism of neural plasticity is complex. This issue has been reviewed in detail for the process of long-term potentiation of synaptic strength (Sanes and Lichtman, 1999). Obstacles to be overcome include the heterogeneity of the process of epileptogenesis. For example, Rasmussen’s encephalitis or anti-NMDAR encephalitis seem unlikely to entail precisely the same molecular pathophysiology as post-traumatic epilepsy. Nevertheless, inflammation has been strongly implicated in prior studies of epilepsy associated with hippocampal sclerosis (Ravizza et al., 2008), which is not considered to be an immune-mediated injury. However, more recent studies have not supported a necessary role for inflammation in hippocampal sclerosis (Aalbers et al., 2014). Another problem is distinguishing whether a system modulates or mediates (i.e., is necessary for) a mechanism of plasticity. Mechanisms of plasticity that involve multiple large networks, each of which is comprised of many types of cells and synapses, are particularly prone to these interpretational difficulties.

The interpretation of experimental results is further complicated because some mediators of the inflammatory response are also involved in physiological neural plasticity. Thus, when these inflammatory mediators are blocked, the subsequent effects on epilepsy are often interpreted to be a consequence of the effects of the blockade on the inflammatory system. However, it is equally possible that the effects on epilepsy are a consequence of the blockade’s interference with normal mechanisms of synaptic plasticity. This issue is more easily appreciated when considered for systems that are anatomically separated. For example, the gastrointestinal system is not considered to be a mediator of neural plasticity because of the effects of antagonists of vasoactive intestinal polypeptide or cholecystokinin on epileptogenesis (Dobolyi et al., 2014). The effects of IL-1β, IL-6, and TNFα antagonists on epileptogenesis provide the same strength of evidence that the inflammatory system is a key mediator of epileptogenesis.

One approach to these interpretational difficulties is to remove as many of the “upstream” elements of inflammation as possible and measure the effects on epileptogenesis. Based on our observations using the *in vitro* organotypic hippocampal slice culture model of post-traumatic epileptogenesis, cellular elements of the immune and inflammatory systems were not necessary for epileptogenesis. We look forward to other laboratories’ dissection of this problem *in vivo* and *in vitro*, including focused genetic deletion experiments with wild-type littermate controls. Because microglia are considered to be the final common phagocytic pathway of the brain’s inflammatory response (Schafer et al., 2013), we did not extend these experiments to consider the effects of antagonists of individual chemical mediators of the inflammatory response. Such experiments would be difficult to interpret for the reasons described above. Thus, the immune and inflammatory systems may modulate, but do not mediate, at least some forms of epileptogenesis.

In the epilepsy syndromes in which the involvement of the immune and inflammatory systems appear more obvious, such as Rasmussen’s encephalitis and NMDAR antibody encephalitis, we do not yet know whether the ongoing activity of these systems is necessary, or whether they are involved in a temporally restricted number of stages of epileptogenesis, and are not subsequently necessary once those stages are complete. For example, in Rasmussen’s encephalitis, anti-inflammatory therapy is ineffective (Freeman, 2005), indicating that at some point in the process of epileptogenesis, epilepsy becomes independent of the inflammatory system. In contrast, NMDAR antibody encephalitis is often responsive to immune therapy (Titulaer et al., 2013).

In light of the current results and the effects of anti-inflammatory and immune therapies on human and experimental epilepsies, the role of inflammatory mediators in epilepsy can be interpreted in three ways. First, there are mechanisms of epileptogenesis that are independent of the immune and inflammatory systems, as demonstrated here. Second, there are epilepsy syndromes for which the immune and/or inflammatory system may be a necessary component of at least some stages of epileptogenesis, although direct evidence for this has not yet been developed. Third, the inflammatory system may not contribute directly to epileptogenesis, but manipulation of the shared mediators of synaptic plasticity and inflammation may produce beneficial anticonvulsant or antiepileptogenic effects.

### Future directions

Developing simplified *in vitro* models of epilepsy syndromes in which the immune system is suspected to play a central role, such as NMDAR antibody encephalitis, will permit further testing of the role of immunity in epilepsy. Such studies might address a question raised by the current results: if inflammation is not necessary for epileptogenesis, does it nevertheless accelerate or otherwise modulate epileptogenesis? Regardless of the role of the inflammatory system in epilepsy, it will be important to pursue the anticonvulsant and antiepileptogenic effects of the antagonists of shared mediators of inflammation and synaptic plasticity, including cyclooxygenase products, IL-1β, IL-6, and TNFα.

## Synthesis

The decision was a result of the Reviewing Editor Marlene Bartos and the peer reviewers coming together and discussing their recommendations until a consensus was reached. A fact-based synthesis statement explaining their decision and outlining what is needed to prepare a revision is listed below. The following reviewer agreed to reveal his identity: Uwe Heinemann

### Reviewer 1:

This study tests if microglia (and to some extent T lymphocytes) contribute to the formation of epileptiform activity in organotypic slice culture. The data are largely fine, but I have several issues with presentation, interpretation and statistical analyses.

First, the title is completely uninformative and should be changed to something that actually describes the results. On a related note, there is a general tendency throughout the paper to over-interpret the results. The authors only test the role of microglia in a particular (much reduced) model of epileptogenesis. It seems a stretch to generalize conclusively from this model to epileptogenesis in vivo, or even to trauma-induced epileptogenesis in vivo.

Second, the authors only seem interested in anticonvulsant effects of depletion (title p7) yet their data suggest that microglia depletion increases seizures. The authors ignore this (although it seems to only be true for the rat slices not the mouse ones).

Third, I really don't understand what is going on in Fig 5. The % epileptic slices is marked as significant in (A), yet there is no mention of this in the text, or legend, or any information on the stats. It would seem that WT and nude mice should be directly compared - the nude mice have far fewer seizures, of much shorter duration, and many fewer epileptic slices. This seems worth discussing, particularly if trying to exclude influence of lymphatic cells on this process.

The statistics don't seem correct for several measures. One-way ANOVAs are done when often it seems that a 2-way ANOVA would be more appropriate. Secondly, the chi-sq test is very unlikely to detect any difference given the n's used here. I don't believe meaningful conclusions can be drawn from this test. The stats done for Fig 6 are completely unclear. Finally, full statistical information should be provided, particularly for the ANOVAs.

### Minor points -

Why is the mTOR pathway brought up in the intro? This seems quite an aside, as does the material on cell loss.

For fig 3, values are given in the text (p8) only for the depleted slices, not the control slices. Give all or none of the values. On the figure itself, the labels are poor - instead of a/b/c, one could simply label the columns by DIV.

It seems more common to have control values on the left, and experimental on the right in bar graphs. There is also inconsistent ordering of the graphs from figure to figure.

### Reviewer 2:

The present paper investigates the role of the immune system in generation of epilepsy in organotypic hippocampal slice cultures. These develop spontaneous seizure like events and the preparation is considered to be a model of traumatic epilepsy although traumatic epilepsy in the age of neonates is rare and usually affects more white than grey matter. The authors find that time course of epileptogenesis is similar in slices depleted from microglia and from lymphocytes and conclude that at least cellular components of the immune system are not required for epileptogenesis. This is an important finding and may focus the attention more on the oter part of the innate immune system namely the astrocytes.

Critique: Depletion of microglia may not affect perivascular cells which are macrophages protecting the Virchow-Robin space. Chemokines and cytokines from astrocytes may still be important for epileptogenesis. This should be discussed in more detail

An important control is lacking namely to determine whether epileptiform activity was initially due to the presence of gentamycin? See work by I. Langmoen 1993

It would be helpful if the number of slice cultures and the n of animals and te incidence of ictal like activity is given. Personally I do not like ictal like for limbic seizures as they can be restricted to automatisms or just auras which nevertheless are considered a seizures. I would therefore prefer seizure like event instead of ictal like activity

The staley lab compared LDH vs PI as a measure of cell death and found that they did not correlate to well. This should be discussed as a limitation of the study also in relation to studies on organotypic cultures and cells death by the Heinemann lab.

A recent paper by Papgiorgiou related cell death and microglial activation in organotypic slice cultures. This work may be considered by the authors.

There are some language errors and the paper requires language and grammar edition.

### Minor:

Page 2 line 37 Please reformulate the sentence

Page 2 line 43 please reformulate sentence:

Page 3 line 62/63 is necessary for ...

Page 4 Line 75 Quote Gutnick et al 1989 for description of epileptiform activity and eventually for hippocampal slice cultures Albus and colleagues 2013

Page 5 line 108: Regarding effects of clodronate one might give reference to the early work of Hailer et al 2003 and 2004 who also alluded to effects on secretion of chemokinses and of proliferation of astrocytes and was probably the first to describe depletion of microglia from explants cultures.

Page 5 line 114 reformulate sentence

Page 9 I guess a better reference to Lymphocytes and role in epileptogenesis is Fabene and colleagues

## Author Response

### Reviewer 1:

This study tests if microglia (and to some extent T lymphocytes) contribute to the formation of epileptiform activity in organotypic slice culture. The data are largely fine, but I have several issues with presentation, interpretation and statistical analyses.

First, the title is completely uninformative and should be changed to something that actually describes the results. On a related note, there is a general tendency throughout the paper to over-interpret the results. The authors only test the role of microglia in a particular (much reduced) model of epileptogenesis. It seems a stretch to generalize conclusively from this model to epileptogenesis in vivo, or even to trauma-induced epileptogenesis in vivo.

A descriptive title is difficult due to the number of elements of the inflammatory system that were removed: systemic immune and inflammatory mediators, blood-brain-barrier, microglia, and T-lymphocytes. Our first choice for a new title would be (modifications in red):

“What elements of the inflammatory system are necessary for epileptogenesis in vitro?”

A second choice could be

“Epileptogenesis in vitro in the absence of cellular elements of the inflammatory system”

Second, the authors only seem interested in anticonvulsant effects of depletion (title p7) yet their data suggest that microglia depletion increases seizures. The authors ignore this (although it seems to only be true for the rat slices not the mouse ones).

As the referee points out, the proconvulsant effects of microglial depletion were interesting but the magnitude of the effects were modest and not consistent between all strains and species. The section of the Discussion addressing this issue has been revised for clarity (lines 310-313).

Third, I really don't understand what is going on in Fig 5. The % epileptic slices is marked as significant in (A), yet there is no mention of this in the text, or legend, or any information on the stats. It would seem that WT and nude mice should be directly compared - the nude mice have far fewer seizures, of much shorter duration, and many fewer epileptic slices. This seems worth discussing, particularly if trying to exclude influence of lymphatic cells on this process.

The referee is correct, we did not compare WT to nude mice in detail despite apparent differences in rate of epileptogenesis. The rationale is now discussed (Lines 280-281; 304-309)

1) nude mice were not developed on the C57BL/6 background, and wild type controls are not available (Jackson suggests using heterozygous mice: http://jaxmice.jax.org/strain/007850.html).
2) Mice strains exhibit heterogenous rates of epileptogenesis (Papandrea et al. 2009).
3) Slice cultures from nude mice developed epilepsy at the same rate as slice cultures from rats
Thus we feel confident that we can state: epileptogenesis proceeds in the absence of T-lymphocytes. However we cannot make finer comparisons effects on rate of epileptogenesis. If the referee prefers we can perform additional experiments using slices cultures from heterozygous nude mice.

The statistics don't seem correct for several measures. One-way ANOVAs are done when often it seems that a 2-way ANOVA would be more appropriate. Secondly, the chi-sq test is very unlikely to detect any difference given the n's used here. I don't believe meaningful conclusions can be drawn from this test. The stats done for Fig 6 are completely unclear. Finally, full statistical information should be provided, particularly for the ANOVAs.

Additional statistical testing has been performed, including 2-way ANOVAs. The additional information is now included in the statistical table (Lines 266).

### Minor points –

Why is the mTOR pathway brought up in the intro? This seems quite an aside, as does the material on cell loss.

To focus attention on the point that antiepileptogenic interventions do not affect microglial activity, the sentence in the introduction has been revised (lines 61-62) from:

“…the antiepileptogenic effects of mTOR pathway manipulation are not associated with changes in microglial activation (van Vliet et al., 2012).”

to:

“…experimental antiepileptogenic therapies are not associated with changes in microglial activation (van Vliet et al., 2012).”

For fig 3, values are given in the text (p8) only for the depleted slices, not the control slices. Give all or none of the values. On the figure itself, the labels are poor - instead of a/b/c, one could simply label the columns by DIV.

Figure 3 relabeled as suggested, and control values added to page 9 lines 195-196.

It seems more common to have control values on the left, and experimental on the right in bar graphs. There is also inconsistent ordering of the graphs from figure to figure.

Figures (2,3,4 and 5) were modified as suggested

Reviewer 2:

The present paper investigates the role of the immune system in generation of epilepsy in organotypic hippocampal slice cultures. These develop spontaneous seizure like events and the preparation is considered to be a model of traumatic epilepsy although traumatic epilepsy in the age of neonates is rare and usually affects more white than grey matter. The authors find that time course of epileptogenesis is similar in slices depleted from microglia and from lymphocytes and conclude that at least cellular components of the immune system are not required for epileptogenesis. This is an important finding and may focus the attention more on the other part of the innate immune system namely the astrocytes.

Critique: Depletion of microglia may not affect perivascular cells which are macrophages protecting the Virchow-Robin space. Chemokines and cytokines from astrocytes may still be important for epileptogenesis. This should be discussed in more detail

This is an important point. We now specifically state on Lines 115-117 that clodronate also depletes perivascular macrophages at the concentrations below those needed to deplete microglia (Polfliet et al. 2001). These macrophages stain for IBA-1 (Sasaki et al. 2001; Lines 131-132) so they were counted as microglia in our assays.

An important control is lacking namely to determine whether epileptiform activity was initially due to the presence of gentamycin? See work by I. Langmoen 1993

We now address this important point in the Methods lines 97-99:

“This concentration of gentamycin was less than half the lowest concentration known to induce regenerative activity in vitro (Grøndahl and Langmoen 1993).”

n.b. we are submitting a detailed analysis of the effects of antibiotics as well as the myriad other components of culture media on epileptogenesis in this model. This is a separate methods optimization paper that is not referenced here. (We did not find any such components that induced epilepsy).

It would be helpful if the number of slice cultures and the n of animals and the incidence of ictal like activity is given. Personally I do not like ictal like for limbic seizures as they can be restricted to automatisms or just auras which nevertheless are considered a seizures. I would therefore prefer seizure like event instead of ictal like activity

Numbers of animals and slices and incidence of seizure-like activity are summarized in table 1 as suggested (Line 765)

ILA changed to SLE as suggested throughout text

The staley lab compared LDH vs PI as a measure of cell death and found that they did not correlate to well. This should be discussed as a limitation of the study also in relation to studies on organotypic cultures and cells death by the Heinemann lab.

We use LDH to screen for cell death based on the correlation between PI staining and LDH release (Berdichevsky et al. 2012). The section of the Methods regarding lactate and LDH assays has been rewritten for clarity (lines 152-154).

A recent paper by Papgiorgiou related cell death and microglial activation in organotypic slice cultures. This work may be considered by the authors.

Thanks. This paper has been added to the introduction lines 58-59:

Microglial activation after epileptogenic injuries is complex and not strongly correlated with neuronal loss (Papageorgiou et al. 2014).

There are some language errors and the paper requires language and grammar edition.Minor: We appreciate these edits and the text has been changed in all cases as suggested

Page 2 line 37 Please reformulate the sentence done

Page 2 line 43 please reformulate sentence: done

Page 3 line 62/63 is necessary for ...done

Page 4 Line 75 Quote Gutnick et al 1989 for description of epileptiform activity and eventually for hippocampal slice cultures Albus and colleagues 2013 Gutnick et al. 1989 and Albus et al. 2013 added (Lines 77, 79)

Page 5 line 108: Regarding effects of clodronate one might give reference to the early work of Hailer et al 2003 and 2004 who also alluded to effects on secretion of chemokinses and of proliferation of astrocytes and was probably the first to describe depletion of microglia from explants cultures. Kohl…Hailer 2003 reference added as suggested to Introduction (Line 82) and Methods (Line 115)

Page 5 line 114 reformulate sentence done

Page 9 I guess a better reference to Lymphocytes and role in epileptogenesis is Fabene and colleagues done
